# Pemphigoïde bulleuse révélant un carcinome bronchique

**DOI:** 10.11604/pamj.2014.19.45.5160

**Published:** 2014-09-19

**Authors:** Hicham Janah, Meryem Mahhou, Hicham Souhi, Adil Zegmout, Hicham Naji-Amrani, Mohamed Raoufi, Hanane Elouazzani, Ismail Abderrahmani Rhorfi, Ahmed Abid

**Affiliations:** 1 Service de Pneumologie, Hôpital Militaire d'Instruction Mohammed V, Rabat, Maroc; 2 Service de Dermatologie, Hôpital Militaire d'Instruction Mohammed V, Rabat, Maroc

**Keywords:** Pemphigoïde bulleuse, carcinome bronchique, paranéoplasique, Bullous pemphigoid, lung carcinoma, paraneoplastic

## Abstract

La pemphigoïde bulleuse (PB) est la plus fréquente des dermatoses bulleuses auto-immunes, touchants préférentiellement le sujet âgé de plus de 70 ans. L'origine paranéoplasique de La PB est rarement rapportée. Cette lésion peut apparaitre de manière synchrone ou parfois être une manifestation révélatrice de la tumeur. Nous rapportons l'observation d'un jeune patient présentant un cancer bronchique métastatique révélé par une PB. Chez le sujet jeune fumeur, toute pemphigoïde bulleuse justifie la recherche d'une néoplasie.

## Introduction

La pemphigoïde bulleuse (PB) est la plus fréquente des dermatoses bulleuses auto-immunes. L'origine paranéoplasique de la pemphigoïde bulleuse est rarement rapportée. Nous rapportons l'observation d'un jeune patient présentant un cancer bronchique révélé par une PB.

## Patient et observation

Nous rapportons le cas d'un patient âgé de 52 ans, tabagique chronique à raison de 40 paquets-année, qui présentait depuis 3 mois des douleurs thoraciques gauches, des hémoptysies de faibles abondances évoluant dans un contexte de fléchissement de l’état général avec apparition récente des lésions dermatologiques prurigineuses.

**Figure 1:**
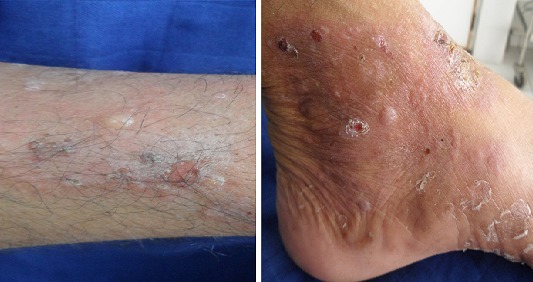
Dermatose bulleuse

**Figure 2:**
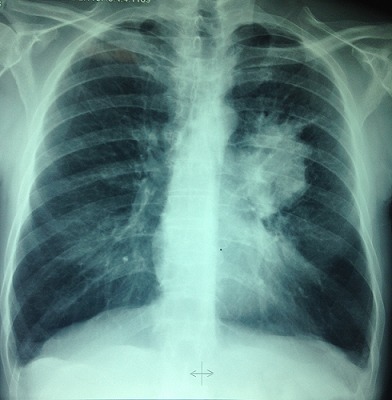
Radiographie thoracique, opacité hilaire gauche

**Figure 3:**
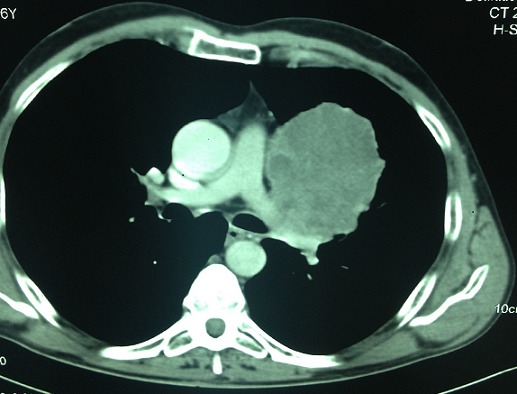
Scanner thoracique, processus tumoral hilaire gauche en contact avec l'artère pulmonaire

**Figure 4:**
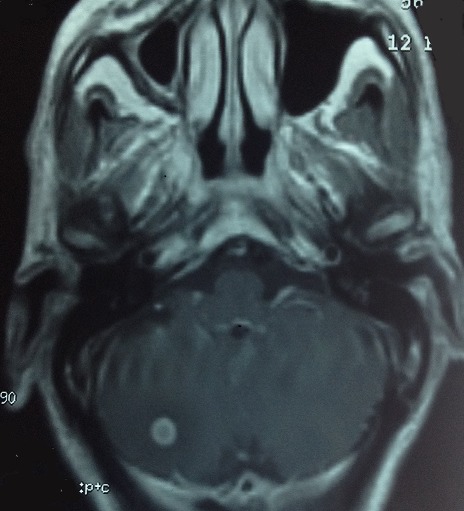
IRM cérébrale, métastase cérébelleuse

L'examen clinique pleuropulmonaire objectivait un syndrome de condensation au niveau de l'hemithorax gauche, l'examen cutané montrait une dermatose bulleuse prurigineuse à contour clair reposant sur des plaques érythémateuse avec un signe de Nikolsky négatif, laissant place à des lésions érosives sans atteinte de la muqueuse buccale ni génitale (Figure 1), intéressant de façon symétrique le tronc et les membres inférieurs faisant évoquer une pemphigoïde bulleuse associée à un cancer bronchique. La radiographie thoracique de face montrait une opacité hilaire gauche, le scanner thoracique objectivait un processus tissulaire nécrotique hilaire gauche, réalisant un contact intime avec l'artère pulmonaire gauche (Figure 2, Figure 3). La fibroscopie bronchique objectivait un bourgeon tumoral blanchâtre obstruant l'orifice du culmen et l’étude anatomopathologique des biopsies revenaient en faveur d'un carcinome épidermoïde moyennement différencié et infiltrant. La biopsie cutanée montrait un décollement sous épidermique avec dépôts de C3 et IgG à l'immunofluorescence directe compatible avec une pemphigoïde bulleuse. Le bilan d'extension du cancer bronchique mettait en évidence une localisation métastatique cérébelleuse (Figure 4). L'hémogramme montrait une éosinophilie modérée. La spirométrie montrait des troubles ventilatoires obstructifs avec un VEMS à 40%.

La décision thérapeutique était de commencer une chimiothérapie à base de cisplatine et navelbine, associée à une radiothérapie thoracique et cérébelleuse et corticothérapie locale. L’évolution a été marquée par la régression des lésions bulleuses après deux cures de chimiothérapie avec amélioration clinique et stabilisation du processus pulmonaire sur un recul de 8 mois.

## Discussion

Les syndromes para-néoplasiques les plus fréquents dans le cancer bronchique sont d'abord l'hippocratisme digital (dans sa forme isolée ou associée à une ostéoarthropathie hypertrophiante), puis les manifestations endocriniennes et neurologiques. Les syndromes para-néoplasiques cutanés sont beaucoup plus rares et regroupent l'acanthosis nigricans, l'acrokératose de Bazex, la dermatomyosite et les dermatoses bulleuses [1]. Les manifestations para-néoplasiques cutanées représentent 7 à 15% des syndromes para-néoplasiques rapportés au cours de l’évolution des cancers [2]. Ces dermatoses para-néoplasiques représentent un groupe hétérogène d'affections qui ne résultent pas de l'extension directe du cancer ou d'une diffusion métastatique. Les manifestations cutanées peuvent précéder, coïncider ou suivre le diagnostic du cancer. L’évolution de la dermatose para-néoplasique accompagne étroitement celle favorable ou non du cancer.

La physiopathologie des dermatoses para-néoplasiques est mal connue: production par la tumeur d'hormones, de decytokines, de facteurs de croissance ou de diverses autres substances encore inconnues; mécanismes immunologiques mettant en jeu des interactions antigènes-anticorps [3]. La pemphigoïde bulleuse (PB) est la plus fréquente des dermatoses bulleuses auto-immunes. Elle représente 70% des dermatoses bulleuses auto-immunes sous-épidermiques avec une incidence annuelle de plus de 400 nouveaux cas par an en France [4, 5]. Elle touche avec prédilection le sujet âgé (âge moyen en France autour de 80 ans). Par ailleurs notre patient était relativement jeune.

Elle se caractérise cliniquement dans sa forme classique par une dermatose bulleuse, prurigineuse, faite de bulles tendues sur plaques érythémateuses, symétriques (faces de flexion des membres, face antéro-interne des cuisses, abdomen), sans atteinte muqueuse ni cicatrice atrophique, sans signe de Nikolsky [6]. L'hémogramme montre fréquemment une hyperéosinophilie sanguine, parfois très importante. Notre patient avait une hyperéosinophilie modérée.

Le diagnostic est affirmé sur la biopsie cutanée avec mise en évidence d'une bulle sous-épidermique à toit respecté en coloration standard, et sur la présence d'anticorps de classe IgG fixés de façon linéaire sur la jonction dermo-épidermique révélés par une technique d'immunofluorescence directe. L'association d'une pemphigoïde bulleuse et d'une maladie cancéreuse fait toujours l'objet de controverse; il existe des rapports faisant état d'améliorations de lésions bulleuses par un traitement anti-tumoral, ce qui pourrait évoquer une relation causale [7]. Dans notre observation, la régression de la dermatose au début de chimiothérapie plaide en faveur du caractère para-néoplasique. Dans le contexte d'une altération de l’état général, la recherche d'une tumeur solide est légitime car l'association avec une pemphigoïde bulleuse n'est pas fortuite, notamment dans les carcinomes pulmonaires et hématologiques [8].

## Conclusion

La pemphigoïde bulleuse paranéoplasique est rare, mais très évocatrice d'une tumeur profonde qu'il faudra rechercher de principe, Son traitement repose essentiellement sur celui de la néoplasie sous jacente.
